# Association between Dietary Habits and Fecal Microbiota Composition in Irritable Bowel Syndrome Patients: A Pilot Study

**DOI:** 10.3390/nu13051479

**Published:** 2021-04-27

**Authors:** Annamaria Altomare, Federica Del Chierico, Giulia Rocchi, Sara Emerenziani, Chiara Nuglio, Lorenza Putignani, Silvia Angeletti, Alessandra Lo Presti, Massimo Ciccozzi, Alessandra Russo, Silvia Cocca, Mentore Ribolsi, Maurizio Muscaritoli, Michele Cicala, Michele Pier Luca Guarino

**Affiliations:** 1 Gastroenterology Unit, Departmental Faculty of Medicine and Surgery, Università Campus Bio-Medico di Roma, 00128 Rome, Italy; giulia.rocchi@unicampus.it (G.R.); s.emerenziani@unicampus.it (S.E.); c.nuglio@unicampus.it (C.N.); silvia.cocca@gmail.com (S.C.); m.ribolsi@unicampus.it (M.R.); m.cicala@unicampus.it (M.C.); m.guarino@unicampus.it (M.P.L.G.); 2 Multimodal Laboratory Medicine Research Area, Unit of Human Microbiome, Bambino Gesù Children’s Hospital, IRCCS, 00147 Rome, Italy; federica.delchierico@opbg.net (F.D.C.); alessandra.russo@opbg.net (A.R.); 3 Unit of Parasitology and Multimodal Laboratory Medicine Research Area, Unit of Human Microbiome, Department of Diagnostic and Laboratory Medicine, Bambino Gesù Children’s Hospital, IRCCS, 00147 Rome, Italy; lorenza.putignani@opbg.net; 4 Unit of Clinical Laboratory Science, University Campus Bio-Medico of Rome, 00128 Rome, Italy; s.angeletti@unicampus.it; 5 Department of Infectious Diseases, Istituto Superiore di Sanità, Viale Regina Elena 299, 00161 Rome, Italy; alessandra.lopresti@guess.iss.it; 6 Unit of Medical Statistics and Molecular Epidemiology, Departmental Faculty of Medicine and Surgery, Università Campus Bio-Medico di Roma, 00128 Rome, Italy; m.ciccozzi@unicampus.it; 7 Department of Translational and Precision Medicine, Sapienza University of Rome, 00185 Rome, Italy; maurizio.muscaritoli@uniroma1.it

**Keywords:** irritable bowel syndrome, microbiota, dietary habits, nutrient intake, Mediterranean diet

## Abstract

Intestinal dysbiosis seems to play a role in the pathophysiology of irritable bowel syndrome (IBS). The present pilot study aimed to elucidate the association between nutrient intake and Mediterranean diet (MD) adherence with IBS symptoms and gut microbiota in IBS patients. The nutrient intake of 28 IBS patients and 21 controls was assessed through a food diary, the reference intake ranges (RIs) for energy-yielding macronutrients and the MD serving score (MDSS) index. MD adherence and nutrients intake were compared to IBS symptoms and fecal microbiota, obtained by 16S rRNA targeted-metagenomics. In IBS patients MDSS index was altered compared to controls (*p* < 0.01). IBS patients with low-MD score reported severe abdominal pain and higher flatulence point-scales. Through Linear discriminant analysis effect size (LEfSe), Erysipelotrichaceae were detected as a microbial biomarker in IBS patients with altered RIs for macronutrients intake, compared to controls. Lactobacillaceae and *Lactobacillus* were associated to an altered carbohydrates intake in IBS patients, while specific taxonomic biomarkers, such as *Aldercreuzia*, Mogibacteriaceae, Rikenellaceae, *Parabacteroides* and *F. prausnitzii* were associated with an adequate intake of nutrient in these patients. This study supports an association between dietary patterns and gut microbial biomarkers in IBS patients. Further investigations are needed to clarify these connections.

## 1. Introduction

Irritable bowel syndrome (IBS) is one of the most common gastrointestinal (GI) functional disorders in the industrialized world, it affects approximately 10–20% of the general population [1]. IBS is characterized by the presence of chronic and recurrent abdominal pain and discomfort associated with changes in stool form and stool frequency, according to the Rome IV criteria [2]. Based on predominant bowel habits, patients with IBS are grouped into three subtypes: diarrhea predominant (IBS-D), constipation predominant (IBS-C) and mixed subtype (IBS-M) with alternating episodes of both diarrhea and constipation [1,2]. The pathophysiology and etiology of IBS is yet unclear. It is a multifactorial disease in which many pathogenic factors seem to play an important role, including altered brain-gut interactions, altered intestinal immunity, increased intestinal permeability, enteric infection/inflammation, genetic predispositions, exogenous and endogenous factors, such as diet and psychosocial factors [3,4,5,6]. In recent years, growing evidence has underlined the potential involvement of the altered gut microbiota in the pathophysiology of several GI disease, such as inflammatory bowel disease (IBD) and IBS [7]. Supporting this hypothesis, several studies have shown an altered gut microbiota composition in IBS patients compared to healthy subjects [8,9], hence influencing the onset of IBS symptoms, such as abdominal pain and flatulence. Differences in the gut microbiota taxonomy of IBS patients have been reported. Decreases in *Bifidobacteria*, *Bacteroidetes* and *Faecalibacterium prausnitzii*, and increases in Firmicutes and Firmicutes/Bacteroidetes ratio are commonly reported [10,11]. Alterations in specific microbial taxa, reduced richness, diversity and temporal instability are reported in IBS patients vs. controls [12], as well as a greater instability in response to dietary changes [13].

Dietary patterns and macronutrient intakes from the diet have a profound impact on gut microbial composition and function and, consequently, on GI symptoms. Specific dietary patterns are able to induce short- and long-term changes in the composition of the gut microbiota, selecting specific bacterial species [14]. Particularly, non-digestible dietary polysaccharides are metabolized through the saccharolytic fermentation processes by gut microbes, such as *Bifidobacteria*, *F. prausnitzii*, *Roseburia*, determining the production of short chain fatty acids (SCFAs) [15,16]. SCFAs exert beneficial and cytoprotective effects on the functional homeostasis of human colonocytes, maintaining the normal integrity of the intestinal epithelial barrier and preventing the entry of antigens into the submucosa with consequent activation of the immune system and low-grade inflammation, detected in some IBS patients. Furthermore, the current evidence suggests an alteration of the intestinal microbiota in IBS, frequently characterized by a reduction in Bifidobacteria species, is associated with a worse symptomatic profile [17]. Proteins represent the major nitrogen source for colonic bacteria. However, fermentation of proteins by the microbiota produces a huge diversity of bacterial metabolites and gases, resulting in exacerbation of GI symptoms, and increased of nitrogenous substrates leads to increases in putrefactive fermentation products [18]. Fats also influence the composition and metabolic activity of the gut microbiota. It has been demonstrated that high-fat diets (HFD) increase blood circulating levels of lipopolysaccharide (LPS), possibly as a consequence of increased intestinal permeability [19], often reported in IBS.

IBS patients attribute their GI symptoms to some food products such as milk and dairy products, legumes, wheat products, artichoke, hot spices, onion, cabbage, and fried foods [20]. Despite the selective choice of food by IBS patients, the intake of calories, fats, proteins and carbohydrates by IBS patients is equivalent to controls, with no difference in the number of meals and meal patterns [21,22,23]. In addition, IBS patients avoid certain foods, some of which belong to the group of low fermentable oligo-, di-, mono-saccharides and polyols (FODMAPs), but have a high consumption of other food products rich in fermentable carbohydrates [23]. Currently, the nutritional strategy for managing IBS symptoms is to restrict them. Several studies consistently demonstrate the clinical efficacy of a low-FODMAP diet in improving both symptoms and quality of life in IBS patients. However, only 50–70% of IBS patients benefit from this approach [24,25,26]. Additionally, a diet low in dietary fiber and plant-based foods (mainly vegetables and fruits), is difficult to maintain for a long time, it can cause deficiencies in vitamins, minerals and natural antioxidants and modifies the intestinal microbiota by significantly reducing the concentration of luminal SCFA-producing bacteria [27].

A balanced diet, such as Mediterranean diet (MD), is important to preserve a greater diversity and complexity of the gut microbiota and the GI tract function [28]. Some evidence supports the hypothesis that MD may also modulate symptoms in GI functional disorders [26,28] and be effective in improving abdominal pain and bloating in patients with IBS, as well as having a higher adherence index [29].

Given the heterogeneity of personal dietary characteristics, GI symptomatology and the diversified nature of the microbiota, the aim of this cross-sectional study was to elucidate the potential associations between MD adherence and macronutrients intake with gut microbiota characteristics and GI symptomatology in an adult population with IBS. This pilot study is an attempt to clarify how the interactions between intestinal microbiota and diet could influence the symptoms of IBS, without the aid of a standard diet conditions.

## 2. Materials and Methods

This cross-sectional pilot study was conducted on patients with a diagnosis of IBS compared to healthy subjects, consecutively enrolled at the Gastroenterology Unit of Campus Biomedico University of Rome from 2015 to 2017 (project: WFR GR-2011-02350817, supported by the Ministry of Health, Italy).

### 2.1. Ethics Statement

All the patients were enrolled after fulfilling the informed consent. The study was performed in accordance with the principles of the declaration of Helsinki and approved by the local ethics committee (Campus Prot. 24/15 PAR ComEt CBM).

### 2.2. Study Population

Since there is no clear evidence in the literature on the underlying mechanisms governing the interactions between dietary patterns, gut microbiota and GI symptoms in IBS, it was not possible to calculate a sample size for the present pilot study.

A gastroenterologist performed a complete clinical and demographic evaluation of controls and IBS patients during the first visit of enrolment.

IBS diagnosis was conducted by using the following diagnostic-therapeutic procedures: (1) clinical evaluation and blood/stool test; (2) questionnaire of intestinal functional disorders, developed according to the Rome IV criteria [30,31]; (3) colonoscopy (RSCS) with multiple biopsies. Patients were excluded for the following criteria: (1) use of antibiotics or probiotic bacterial supplements in the past 3 months; (2) use of nonsteroidal anti-inflammatory drugs (NSAIDs) in the past 3 months; (3) recent diagnosis (less than 3 months) of bacterial or parasitic infections of the GI tract, severe psychiatric disease as the dominant clinical problem, other severe diseases, and a history of drug or alcohol abuse.

Gastrointestinal asymptomatic subjects (using a questionnaire to exclude chronic diseases and any current GI symptoms) were enrolled as controls, with the following inclusion criteria: (1) up to 65 years of age who undergo colonoscopy for colorectal cancer screening; (2) absence of macroscopic lesions (including the presence of diverticula); (3) absence of microscopic lesions evident on the histological examination of colonic biopsy samples taken during the colonoscopy. The exclusion criteria were the same described for IBS patients.

### 2.3. Study Protocol and Sample Collections

All the enrolled patients underwent endoscopic examination of the lower digestive tract conducted to explore the cecum, after preparation with polyethylene glycol (PEG) (4130 L) and waste-free diet the three days prior to endoscopy. All patients collected a stool sample the day before the preparation with PEG. All fecal samples were immediately stored at −80 °C, until processing. All patients completed a symptoms questionnaire, elaborated on the Rome IV criteria, in which the GI symptoms (e.g., abdominal pain and flatulence) were evaluated with a Numeric Rating Scale-11 (NRS-11) [32] for patient self-reporting of pain intensity (0 = no pain, 1–3 = mild pain, 4–6 = moderate pain, and 7–10 = severe pain).

The patients reported all bowel movements in a daily diary for 3 days, based on the Bristol Stool Form scale [33,34]. Based on these details, the stool consistency (average stool consistency/day) and stool frequency (average number of stools/day) were calculated.

Weight and height were measured using standardized techniques by trained clinical staff. Height was measured to the nearest 0.1 cm and weight to the nearest 10 g using digital column scales (SECA, Hannover, MD, USA). The body mass index (BMI) was obtained from an individual’s weight (Kg) divided by their height (m^2^). The BMI classification to define adult person as underweight, normal weight, overweight or obese was used [35]. Dietary data used in this study were obtain from a food diary and were collected by trained dieticians using a 3-day food record conducted on 2 non-consecutive week days and a weekend day, in the week before the stool sample collection. None of the participants had food intolerances and/or food allergies and followed special diets (e.g., weight-reducing or therapeutic diet). Each of the food items and beverages consumed were reported based on the main meals (breakfast, lunch and dinner) and many possible snacking between meals, in order to calculate daily dietary intakes. The diary included details regarding: place and time of meals, ingredients, cooking methods, brand of foods and the food/beverage quantity consumed, expressed in g, mL, domestic measurements (e.g., spoons, teaspoons, cups, glasses, etc.) or standard portions. All subjects were given written instruction to allow for accurate completion of the food registration and instructed to consume their usual diet. Total daily energy (kcal/day) and macronutrient intakes (g/day) were calculated using a computer-aided nutritional analysis program (ProgDieta.exe version Beta 1.3, Italy) based on the Food and Nutrient Composition Tables published by the Council for Agricultural Research and Agricultural Economics Analysis (CREA) [36]. For the calculation of the energy (kcal/g) of proteins, fats and carbohydrates, the program uses the recommendations of Greenfield and Southgate (2003) [37] to express the values as the proportion of energy (E%). Total fiber intake is presented as the average grams of 3 days; each macronutrient is presented as E% average of 3 days and evaluated in relation to the reference intake ranges (RIs) of dietary reference values, considering the IV Revision of LARN 2014 (Reference Levels of Nutrients and Energy Intake for the Italian population) of the SINU (Italian Society of Human Nutrition) [38]. The RI is used for energy-yielding macronutrients. It is expressed as the proportion (%) of energy derived from that macronutrient. RIs represent ranges of intakes that are adequate for maintaining health and are associated with a low risk of selected chronic diseases.

In accordance with the RIs recommendations for carbohydrate (45–60 E%), fat (20–35 E%) and protein (>15 E%), IBS patients and controls were divided into two groups: (1) “non-LARN” for the group outside RIs and (2) “LARN” for the group within RIs. Furthermore, considering the overall intake of all nutrients, a balanced amount of macronutrients intake defined in this study as “Macronutrients (MNs) Group”, was classified considering all the following inclusion criteria: (1) 45–60 E% for carbohydrates; (2) 20–35 E% for lipids; (3) >15 E% for proteins; while an unbalanced amount of macronutrients intake, defined as “Non-Macronutrients (non-MNs) Group”, was classified considering one or more of the following inclusion criteria: (1) a lower (<45 E%)/higher (>60 E%) value for carbohydrates; (2) a lower (<20 E%)/higher (>35 E%) value for lipids; (3) a lower (<15 E%) value for proteins.

A well-trained dietician interviewed participants face-to-face to assess MD adherence. The Mediterranean Diet Serving Score (MDSS) from Monteagudo et al. [39] was used to assess the Mediterranean Diet (MD) adherence degree based on the frequency of consumption of foods and food groups. It is based on Mediterranean Diet Pyramid [40], using the recommended consumption frequency of different foods and food groups that should be consumed in every meal (olive oil, cereals, vegetables and fruits), daily (nuts and dairy products), and weekly (fish, white meat, red meat, eggs, legumes, potatoes and sweets). Subjects whose intake of foods and food groups was within the recommended number of servings were assigned a score of 3, 2, or 1 point for recommendations expressed in times/meal, times/day, or times/week, respectively. A score of 0 was assigned when the number of servings was higher or lower than recommended. Then, 1 point was added for alcohol intake (fermented drinks) equivalent to 2 and 1 glass of wine or beer for males and females, respectively. MDSS varies between 0 and 24 points and a ≥16 points’ score means adherence to the MD [39].

### 2.4. DNA Extraction, Amplification for Pyrosequencing, Statistical Analysis

QIAamp DNA Stool Mini Kit (Qiagen, Germany) was used to manually extract DNA from 200 mg of stool samples, as described previously [41]. The V1-V3 regions (520 bp) of the 16S ribosomal RNA locus were selected to performed the pyrosequencing analysis on a 454-Junior Genome Sequencer (Roche 454 Life Sciences, Branford, USA), according to Ercolini et al. [42]. The microbial libraries were amplified by polymerase chain reactions (PCR) from DNA using a Hi-Fi PCR Taq polymerase (FastStart™ High Fidelity PCR System, dNTPack, Roche Diagnostics, Mannheim, Germany) using barcoded primers (Forward 5′-GAGTTTGATCNTGGCTCAG-3′, Reverse 5′-GTNTTACNGCGGCKGCTG-3′) (Roche 454 Life Sciences, Branford, USA). A Quant-iT PicoGreen dsDNA kit (Life Technologies Corporation, Oregon, U.S.A) was used for PCR amplicons purification, following the manufacturer’s instruction. The bacterial libraries were pooled in equal concentrations prior the sequencing reactions. After pyrosequencing reactions, background signals were subtracted and the sequencing images were normalized and transformed into read flowgrams and basecalls with associated per-base quality scores (GS sequencer software v. 2.7, Roche Diagnostics, Mannheim, Germany) and finally trimmed on the base of ends signal quality (GS sequencer software v. 2.7, Roche Diagnostics). QIIME 1.9.0 software was used to analyze raw sequences [43]. The sequences were demultiplexed and filtered for average quality score, length and ambiguous base calling. Sequences were denoised [44] and singletons were excluded. The denoised sequences were chimera-checked by identify_chimeric_seqs.py using both Blast_fragments and ChimeraSlayer (http://qiime.org/scripts/identify_chimeric_seqs.html) approaches. The operational taxonomic units (OTUs), defined by a 97% similarity, were de novo picked and the representative sequences were submitted to PyNAST for the sequence alignment [38], and UCLUST for sequence clustering [45]. The database for OTUs matching was greengenes (v 13.8). After rarefying, the α-diversity analysis was conducted by alpha_rarefaction.py. The Shannon index was applied to microbiota richness and a nonparametric test was used in the comparisons of the index among the groups, the *p*-values were calculated by Monte Carlo analysis and the corrected by Bonferroni tests by compare_alpha_diversity.py. The β-diversity tests by unweighted UniFrac metric were carried out by QIIME software using beta_diversity_through_plots.py and plotted by PCoA plot. The nonparametric Adonis analysis was applied on UniFrac distance matrix to determine sample grouping and a *p*-value computed to determine the statistical significance.

The algorithm for high-dimensional biomarker discovery and explanation, based on linear discriminant analysis (LDA) effect size (LEfSe) [46], was employed to identify taxa features that are statistically different among groups. Specifically, this algorithm uses the non-parametric factorial Kruskal–Wallis (KW) sum-rank test followed by Wilcoxon rank-sum test. As the last step, LEfSe uses linear discriminant analysis to estimate the effect size of each differentially abundant feature.

All sequencing data associated with this study were uploaded to the NCBI bioproject database: PRJNA391149 (https://www.ncbi.nlm.nih.gov/bioproject/?term=PRJNA391149).

### 2.5. Statistical Analysis for Nutritional and GI Data

All dietary and symptoms results were expressed as the mean value; standard deviation, median and range were calculated with conventional methods. Intergroup comparisons of continuous variables that were normally distributed were calculated with the independent samples Student’s *t*-test. Data that were not normally distributed were tested with the Mann–Whitney U test. Normality of data were assessed by the Shapiro-Wilk test and the Kolmogorov-Smirnov test. Statistically significance was considered at a value of *p* < 0.05 for all tests.

The odds ratios (ORs) and 95% confidence intervals (CIs) were estimated to compare the relative impact of dietary habits on the intensity of IBS-symptoms, such as abdominal pain and flatulence, calculated with multinomial logistic regression adjusting for age, gender, body mass index (BMI) and energy.

Software package GraphPad Prism Version 8.4.1 for Windows (GraphPad Software, San Diego, CA, USA) was used to perform statistical analyses.

## 3. Results

### 3.1. Study Population

A total of 28 IBS patients and 21 controls were recruited at the Gastroenterology Unit of the Campus Bio-Medico Hospital (Rome, Italy) from 2015 to 2017. The median age of the study population was 55 years for the IBS group and 56 years for the control group. Males represented 32% and 43% of IBS patients and controls, respectively.

After classification according to Rome IV, 8 subjects (29%) were affected by IBS-M, 11 subjects (39%) by IBS-D and 9 subjects (32%) by IBS-C. All IBS patients reported abdominal pain and flatulence at least once daily. IBS symptoms were recorded according to the NRS-11; results are shown in Table 1, with the demographic and clinical characteristics of IBS patients and of controls. The GI symptoms were markedly increased compared to healthy controls.

### 3.2. Dietary Habits

Studying the food diaries of both groups, no statistically significant difference was observed for the daily calories, carbohydrates, lipids, proteins and total fibers intake in both patients and controls. Interestingly a significant difference was found between IBS patients and controls for the adherence to the MD, assessed through the MDSS (*p* < 0.01). The dietary characteristics of IBS patients and controls are shown in Table 2.

The food frequency evaluation showed that the most IBS patients follow an unbalanced diet, with reduced intake of vegetables (*p* < 0.05), walnuts (*p* < 0.01), milk and dairy products (*p* < 0.05), fish and seafood (*p* < 0.01). Food frequency intake, in accordance with the MD characteristics, are shown in Supplementary Table S1.

The division of IBS patients and controls into the LARN and non-LARN groups for carbohydrates, lipids, proteins and into the MNs and non-MNs groups, according to the LARN 2014 of the SINU, didn’t show significant differences, except for lipid intake (*p* = 0.003) (Table 3) in which only 5 (24%) of the controls had a mean total lipid consumption inadequate to LARN recommendations, compared to 19 (68%) in the IBS group. This difference could be justified by the fact that IBS group stated that they do not regularly consume fish and seafood, milk and dairy products and walnuts, as described previously.

### 3.3. Association between Dietary Habits and Symptoms in IBS Patients

By observing the eating habits of IBS patients and the abdominal pain and flatulence occurring in the post-prandial period, the association between the severity of GI symptoms and the quality of the diet was explored. The foods most often associated with GI symptoms, as reported in the food diaries, were bread (both gluten-free and gluten-containing), pizza, desserts or cakes, legumes, vegetables (such as chicory, asparagus, artichokes, fennel), cow’s milk, fruits (such as orange), salami, fried foods, sauces, coffee, sugary drinks and alcohol (data not shown).

Table 4 shows the relationship between current nutrients intake and IBS severity symptoms, such as abdominal pain and flatulence, assessed by multinomial logistic regression. The adjusted ORs for mild abdominal pain and flatulence is 1.75 times higher for IBS patients who have a high-MD adherence degree, compared to IBS patients who don’t follow MD recommendations (OR = 1.75; 95% CI: 0.73–41.86). Unfortunately, the comparison between IBS-symptoms and dietary habits in IBS patients didn’t show statistically significant differences.

### 3.4. Faecal Sample Collections

A total of 46 fecal samples were analyzed in this study, 26 from IBS patients and 20 from healthy subjects. A total of 136,692.00 sequencing reads were obtained from the 46 fecal samples with a median value of 2833.00 reads. A total of 2 IBS patients and 1 control were excluded from the analysis because no sequences were obtained from stool samples during the analysis.

### 3.5. Microbiota Features Associated to Dietary Habits

The IBS patients were subgrouped on the bases of carbohydrate, fat and protein ranges intake and analyzed for both α- and β-diversity. These analyses did not highlight statistical differences for both diversity parameters (Supplementary Figure S1).

**Figure 1:**
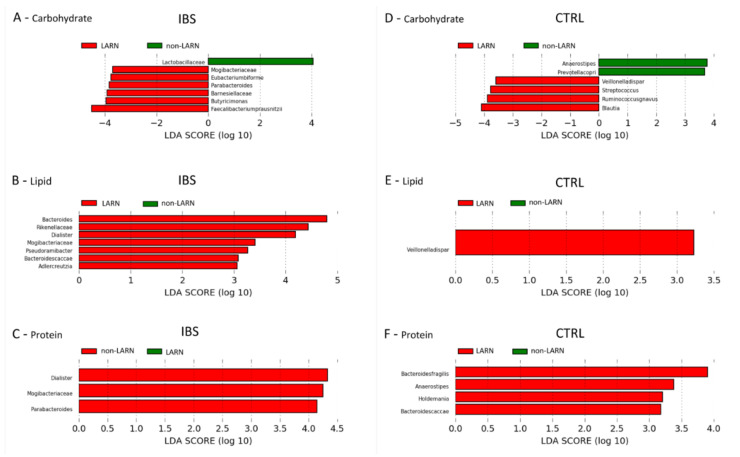
LEfSe analysis of taxonomic biomarkers of gut microbiota in IBS patients and in control subjects. (**A**) IBS samples grouped by carbohydrate intake; (**B**) IBS samples grouped by fat intake; (**C**) IBS samples grouped by protein intake; (**D**) control samples grouped by carbohydrate intake; (**E**) control samples grouped by fat intake; (**F**) control samples grouped by protein intake.

By comparing the microbiota of patients grouped on the bases of carbohydrates % E consumption, we obtained an increment of Mogibacteriaceae, *Eubacterium biforme*, *Parabacteroides*, Barnesiellaceae, *Butyricimonas* and *F. prausnitzii* for the LARN group, while, Lactobacillaceae were incremented in non-LARN group (Figure 1A).

By considering the fats intake, an increment of *Bacteroides*, Rikenellaceae, *Dialister*, Mogibacteriaceae, *Pseudoramibacter*, *Bacteroides caccae* and *Adlercreutzia* in LARN group was detected (Figure 1B). In addition, subgrouping IBS patients on the bases of protein intake an increment in relative abundances of *Dialister*, Mogibacteriaceae and *Parabacteroides* in LARN group was detected (Figure 1C).

For the control population, we didn’t observe statistical differences for both α- and β-diversity patterns with respect to carbohydrate, fat and protein RIs’ adherence of the subjects (Supplementary Figure S2).

By analyzing the carbohydrates consumption, *Veillonella dispar*, *Streptococcus*, *Ruminococcus gnavus* and *Blautia* appeared increased in LARN group; while in non-LARN group *Anaerostipes* and *Prevotella copri* were incremented (Figure 1D). In lipid LARN group Veillonella dispar was increased (Figure 1E), while in protein LARN group were increased *Bacteroides fragilis*, *Anaerostipes*, *Holdemania* and *Bacteroides caccae* (Figure 1F).

### 3.6. Comparison between the Microbiota Profiles of IBS vs. Control LARN Group for Carbohydrate, Fat and Protein Intake

**Figure 2:**
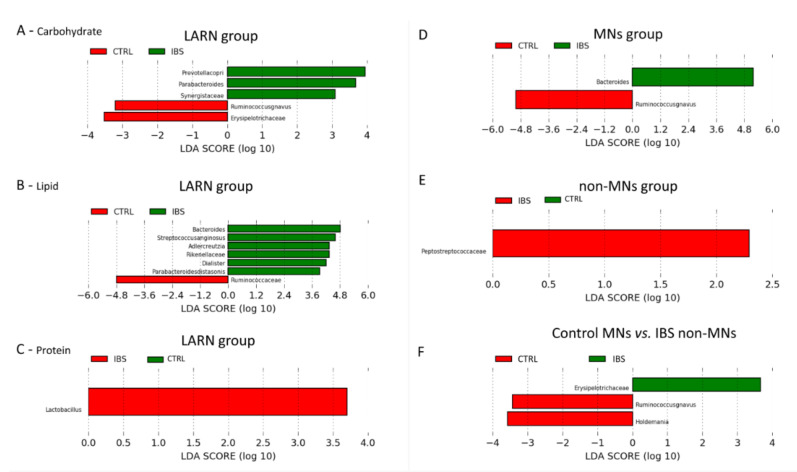
LEfSe analysis of taxonomic biomarkers of gut microbiota of IBS vs. control for carbohydrate, fat, protein and all macronutrients intake. (**A**) Comparison between IBS vs. control samples grouped by carbohydrate intake (LARN group); (**B**) comparison between IBS vs. control samples grouped by lipid intake (LARN group); (**C**) comparison between IBS vs. control samples grouped by protein intake (LARN group); (**D**) comparison between IBS vs. control samples grouped by all macronutrients intake (MNs group); (**E**) comparison between IBS vs. control samples grouped by macronutrient intake (non-MNs group); (**F**) comparison between IBS non-MNs group vs. control MNs group.

Selecting individuals from the LARN group of each 3 macronutrients, we performed a comparison between IBS patients and controls. For all classes of macronutrients, we did not obtain statistical differences by comparing α- and β-diversity parameters (Supplementary Figure S3). By analyzing carbohydrate intake, in controls were increased *R. gnavus* and Erysipelotrichaceae, while in IBS patients were increased *Prevotella copri*, *Parabacteroides* and Synergistaceae (Figure 2A). In addition, analyzing fat intake an increment of *Bacteroides*, *Streptococcus anginosus*, *Adlercreutzia*, Rikenellaceae, *Dialister* and *Parabacteroides distasonis* was observed in IBS group, while Ruminococcaceae were increased in controls (Figure 2B). Additionally, the protein intake analysis revealed an increase of *Lactobacillus* in IBS patients (Figure 2C).

### 3.7. Comparison between the Microbiota Profiles of IBS vs. Control MNs Group and Non-MNs Group

By considering only subjects of MNs group and non-MNs group for all macronutrients, we performed a comparison between IBS and Controls. For α- and β-diversity analyses statistical differences were not observed (Supplementary Figure S4). For MNs group, *Bacteroides* was increased in IBS, while *R. gnavus* was increased in control (Figure 2, Panel D). In non-MNs subjects, an increase of Peptostreptococcaceae was observed in IBS group (Figure 2, Panel E). Comparing the MNs group of controls and non-MNs group of IBS an increase of *R. gnavus* and *Holdemania* was detected in controls, while a significant increase of Erysipelotrichaceae was observed in IBS patients (Figure 2, Panel F). Between these two subgroups no statistical differences were obtained regarding α-and β-diversity comparisons (Supplementary Figure S4)

## 4. Discussion

The present study aimed to investigate the current dietary characteristics, adherence to the MD and the daily intake of macronutrients in an adult study group with IBS, compared to controls, in order to evaluate the adequacy of the diet. Moreover the intensity level of GI symptoms and gut microbiota alterations were explored in these patients, grouped according to the reference intake ranges (RIs) for macronutrients. In accordance with EFSA dietary reference values [47] and SINU guidelines [38], the RIs for macronutrients, expressed as % of energy intake, indicate the amount of a single nutrient that people need to maintain good health depending on their age and gender and associated with a low risk of chronic diseases. Using these recommendations to evaluate the level of nutrients intake and the adequacy of their usual diet, the majority of IBS patients exhibited a significant difference for total lipids consumption (68% IBS vs. 24% controls), outside RI, compared to the control group, mainly linked to a significant limited consumption of fish, seafood and nuts reported in the present study. These results are in accordance with previous findings [21,48,49,50] in which most of IBS patients showed a trend of displacement in energy and nutrients intake compared with an age- and gender-matched control group. The inadequacy of the diet, with intakes above (or below) the lower and upper limits of the acceptable range of each macronutrient, is supported by the results obtained for MD-adherence, which was significantly lower in IBS patients than in controls. Non-compliance with some recommendations of MD-dietary patterns frequency intake, such as fruits, vegetables, legumes, fish and seafood, milk and dairy products, desserts (sugar, pastes, sugary drinks, etc.) and walnuts was detected, although not fully supported by a statistical analyses difference. These findings could be explained by the fact that many of the IBS patients report that their symptoms are triggered by specific foods–most commonly implicated milk and milk products, wheat products, some fruits and vegetables, cabbage, onion, peas/beans, caffeine, certain meat, hot spices, fried food and smoked products–limiting or excluding them from their usual diet [20,51]. Many of these foods contain a number of fermentable carbohydrates with prebiotic effects [17], such as inulin-type fructans (fructo-oligosaccharides, inulin, oligofructose) and galacto-oligosaccharides (GOS), which are generally poorly absorbed in the small intestine and fermented by gut saccharolytic bacteria, causing gas production, distension of the large intestine with abdominal discomfort or pain [20,23]. However, it is not clear whether they are really the cause of this disease, but their restriction might lead to specific changes in the composition and/or activity of gut microbiota and a reduction in SCFAs-producing bacteria [17,24,25,27], with possible adverse effects on GI symptoms. Furthermore, it is known that a high-fat diet (HFD), such as Western diet (WD), has been strongly related to changes in the gut microbiota. The gut microbiota of a WD is characterized by a significant reduction in microbial diversity, species richness and a significant reduction of bacterial species producing SCFAs [26,52]. Therefore these alterations could contribute to the processes of low-grade inflammation in patients with several functional GI disorders [53,54].

Although no statistical differences were observed for both α- and β-diversity parameters, it is interesting to note that specific bacterial biomarkers were associated to IBS patients with an altered diet. In particular, Lactobacillaceae and *Lactobacillus* seemed to be associated to an inadequate consumption of carbohydrates; Erysipelotrichaceae of the phylum Firmicutes were linked to IBS with non-MNs profile, compared to control MNs group. An increase of *Lactobacillus* genus has been correlated with high production of L- and D-lactate when the carbohydrates of the diet are in excess and are not completely absorbed [55,56]. In this scenario, an abnormal production of lactate and pH acidification of the colon causes a greater proliferation of bacteria responsible for lactic acid synthesis, mainly *Lactococcus*, *Streptococcus*, *Lactobacillus fermentum* and *Lactobacillus acidophilus* [57] that, however, also have mucolytic activity, altering the epithelial intestinal barrier. Interestingly, in a study conducted in cats, class Erysipelotrichi and genus *Lactobacillus* were increased in feces from cats with chronic diarrhea [58], suggesting their potential involvement in the intestinal functional impairment. Moreover, our data agree with multiple targeted metagenomics analyses in which the abundance of Erysipelotrichi is strongly associated with a WD and after HFD treatment [59,60]. In this context, it has been hypothesized that a diet not adequately distributed in the daily intake of carbohydrates, fats and proteins could produce a direct effect on some specific taxa recognized to be closely correlated to adiposity, colorectal cancer and interleukin (IL)-1β levels [61,62], possibly promoting a pro-inflammatory intestinal state that could alter intestinal permeability in IBS patients.

By investigating the gut microbiota of IBS patients with a balanced intake of macronutrients, specific taxonomic biomarkers were associated to the group with adequate RI for lipid, such as *Adlercreutzia*, and Rikenellaceae; while in the group within RI for carbohydrate consumption Mogibacteriaceae, *Parabacteroides* and *F. prausnitzii* were detected. A study conducted on healthy adults showed that Mogibacteriaceae and Rikenellaceae were positively correlated with a high frequency of bowel movements [63], suggesting that in IBS-C patients, the frequency of bowel movements could be controlled by modulating the abundance of Mogibacteriaceae and Rikenellaceae through a balanced diet that satisfies the dietary recommendations for carbohydrate and fat intake.

*F. prausnitzii* is one of the most abundant anaerobic bacterial species that is becoming recognized as an important marker for gastrointestinal health and important for the maintenance of gastrointestinal health [64]. Moreover, it is able to metabolize complex carbohydrates from the diet producing butyrate, which represents an important source of energy for colonocytes and prevents the invasion of pathogens by strengthening the intestinal barrier. Butyrate also participates in immune modulation reducing the expression of pro-inflammatory cytokines (e.g., IL-8, interferon [INF]-γ and tumor necrosis factor [TNF]-α) and stimulating the production of anti-inflammatory cytokines (e.g., IL-10 and IL-12) generally decreased in patients with a WD and a reduction of dietary fibers [65,66]. These anti-inflammatory properties can protect the colon from the inflammatory processes, and a significant loss of *F. prausnitzii* has been associated with a change in the microbiota of patients with different chronic GI disorders [67,68]. Therefore, most IBS patients could (and should) follow a balanced diet with a well-balanced intake of macronutrients, without restrictions and/or excesses, and without concentrating the intake of fermentable sugars in a meal or in a limited period. The modulation of the gut microbiota, through an adequate intake of prebiotics and dietary fiber, could significantly increases the abundance of beneficial commensals, improving the GI symptoms severity and the intestinal function in subjects with several intestinal disorders. These results were already reported for healthy subjects that followed the consumption of prebiotics (inulin 10 g/day) or dietary fibers (21 g/day) [69,70].

Moreover, the LEfSe analysis in the present study has identified some taxa strictly associated to healthy subjects. For instance, *V. dispar* was linked to the LARN group of fats and carbohydrate consumption and *R. gnavus* to both MNs-group and LARN group of carbohydrate. Interestingly, *R. gnavus* is known to produce an antibacterial peptides and SCFAs [71], both with protective effects in the host from the pathogens. *Bacteroides*, *Bacteroides fragilis* and *Bacteroides caccae* were increased both in IBS and controls group with an adequate % energy of lipid and protein intake. *Bacteroides* spp. are considered important bacteria in maintaining intestinal health, because they strengthen the epithelial barrier and produce anti-inflammatory molecules such as polysaccharide A (PSA), sphingolipids and outer membrane vesicles (OMV) [72,73]. In a recent study, it was observed that *B. caccae*, *B. intestinalis* and *B. vulgatus* significantly reduced IL-8 levels in a LPS-induced enterocyte cell line, demonstrating their in vitro ability to attenuate inflammation [74]. In addition, *B. fragilis* has been shown to relieve LPS-induced inflammation in mouse models by decreasing TNF-α, increasing IL-10 cytokines and restoring the Treg/Th17 balance [75,76]. Further investigations are needed to evaluate the safety of *Bacteroidetes* spp., because of some strains are also considered opportunistic pathogens which may induce the pro-inflammatory processes and play a role in the pathogenesis of chronic GI diseases [77,78].

## 5. Strengths and Limitations of the Study

According to the current knowledge, this is one of the first studies aiming to explore the possible associations of MD adherence and macronutrient intake with the gut microbiota profile and GI symptoms prevalent in adult IBS patients. The results of the present investigations note that lower severity of GI symptoms in the IBS group, such as flatulence and abdominal pain, appeared to be associated with higher MD adherence, compared to those who had a poor level of adherence to MD recommendations. In addition, the subdivision of IBS patients and controls by LARN and non-LARN groups showed a significant difference in total dietary fat intake, demonstrating a higher prevalence of IBS patients in not satisfy the RI for lipid.

Certainly, the reported results should be considered in light of some limitations: (1) this study is based on a modest size cohort and requires a more significant number of patients, in further investigations, to corroborate these preliminary data; (2) there was a loss of three fecal samples because no sequences were obtained during the analysis; (3) it was not possible to carry out comparative analysis relating to dietary fiber intake as both groups had a negligible average daily intake; (4) the study population is biased towards females, this may have led to bias, but is a direct representation of the demographic characteristics of the IBS patients to whom our department refers; (5) the 454 platform for microbiota pyrosequencing used in this study, results currently overcome by more performing technologies. However, during the developing of this project the 454 platform was widely used in microbiota studies. The limited patient size allows only a first description of the relationship between diet, fecal microbiota and GI symptoms, but the sample size and the fact that this is a case-control study preclude definitive identification of the causal link. At the same time, the preliminary conclusions of this study invite further exploration of these aspects in future research, with a larger patient’s number.

## 6. Conclusions

In conclusion, the underlying mechanisms governing the interaction between dietary patterns, gut microbiota diversity and the host are still unclear in IBS. The available data on the MD, GI symptoms and modulation of the gut microbial structure in IBS patients are rather scarce. Based on the present investigation, the adherence to MD recommendations is associated with a lower risk of severe GI symptoms. Furthermore, IBS patients who met macronutrient RIs were characterized by increased colonization patterns of SCFA-producing bacteria, such as *F. prausnitzii* and Rikenellaceae. Certainly, the influence of the MD on gut microbial ecology is a scientific field open to further investigation to understand the safety and efficacy of a MD-based dietary strategy in IBS patients. IBS is a heterogeneous entity and the growing knowledge of its pathophysiology supports the potential of dietary therapies to modulate the intestinal microbiota, not only to improve symptoms. More accurate personalized prediction methods need to be developed by combining basic microbiome signatures with other important clinical traits, such as GI symptoms. Therefore, understanding the variations and fluctuations in microbiota profiles and concentrations of host or microbial derived metabolites, could be used to infer the processes that contribute to symptoms onset and severity of IBS, providing important new insights on the treatment of IBS.

## Figures and Tables

**Table 1:** Subject Characteristics.

	Control (*n* = 21)	IBS (*n* = 28)	*p*-Value
Age years, median (range)	56 (65–26)	55 (69–29)	0.92
Sex, Males, *n* (%)	9 (43)	9 (32)	0.44
BMI (Kg/m^2^), mean ± sd	24.99 ± 3.2	27.08 ± 5.2	0.16
Underweight, mean ± sd, (*n*)	-	-	
Normal weight, mean ± sd, (*n*)	22.14 ± 1.59 (8)	22.39 ± 2.02 (11)	0.49
Overweight, mean ± sd, (*n*)	25.79 ± 1.22 (11)	27.63 ± 1.53 (11)	1.0
Obese, mean ± sd, (*n*)	31.95 ± 2.76 (2)	34.67 ± 4.04 (6)	0.15
Predominant Bowel Habits, *n* (%)			
Constipation (IBS-C)	-	9 (32)	NA
Diarrhoea (IBS-D)	-	11 (39)	NA
Mixed (IBS-M)	-	8 (29)	NA
Stool frequency (*n*/day), mean ± sd	1.19 ± 0.68	1.64 ± 1.37	0.17
Stool consistency (BSS), mean ± sd	3.67 ± 0.80	3.79 ± 1.97	0.44
Abdominal pain*, n (*%*)*	3 (14)	28 (100)	**<0.001**
Frequency (*n*/day), mean ± sd	0.24 ± 0.62	2.96 ± 2.03	**<0.001**
Intensity, mean ± sd	0.48 ± 1.21	6.25 ± 1.24	**<0.001**
Flatulence*, n (*%*)*	5 (23)	28 (100)	**<0.001**
Frequency (*n*/day), mean ± sd	0.62 ± 1.2	4.89 ± 2.08	**<0.001**
Intensity mean ± sd	1.05 ± 1.94	7.21 ± 1.17	**<0.001**

Normally or non-normally distributed data were tested with the independent samples Student’s *t*-test and Mann–Whitney *U* test, respectively. The categorical variables were tested with the Chi-squared test. Significant *p*-values (<0.001). NA = not applicable; BMI = body mass index; BSS = Bristol stool scale.

**Table 2:** Dietary Characteristics.

	Control (*n* = 21)	IBS (*n* = 28)	*p*-Value ^a^
Energy, kcal/day	1425 ± 519.5	1484 ± 532.2	0.56
Carbohydrates, g/day (E%)	199 ± 77.6 (49)	174 ± 75.1 (47)	0.29
Lipids, g/day (E%)	60 ± 18.6 (34)	56 ± 19.5 (37)	0.46
Proteins, g/day (E%)	62 ± 21.5 (16)	61 ± 20.1 (16)	0.38
Total fibers, g/day	14 ± 5.1	12 ± 5.8	0.24
MD score ^b^	17 ± 4.9	11 ± 3.7	**<0.01**

Energy and nutrients intake are calculated from food diaries and are expressed as mean ± SD. ^a^ All data no normally distributed were tested with the Mann–Whitney *U* test. Significant *p*-values (<0.01). ^b^ MD score, Mediterranean diet score, evaluated according to the Mediterranean diet serving score (MDSS). A score ≥ 16 indicates adherence to the dietary recommendations of the MD.

**Table 3:** The division of IBS and controls into the “LARN” and “non-LARN” groups.

Groups	Control (*n* = 21)	IBS (*n* = 28)	*p*-Value ^a^
**Carbohydrate intake**			
LARN group, (45–60 E%), *n* (%)	14 (67)	12 (43)	0.15
Non-LARN, (<45–>60 E%), *n* (%)	7 (33)	16 (57)	
**Lipid intake**			
LARN group, (20–35 E%), *n* (%)	16 (76)	9 (32)	**0.003**
Non-LARN group, (<20–>35 E%), n (%)	5 (24)	19 (68)	
**Protein intake**			
LARN group, (>15 E%), *n* (%)	12 (57)	18 (64)	0.79
Non-LARN group, (<15 E%), *n* (%)	9 (43)	10 (36)	
**All Macronutrients Intake**			
MNs group, *n* (%)	7 (33)	3 (11)	0.07
non-MNs group, *n* (%)	14 (67)	25 (89)	

^a^ Data were tested with the Fisher’s exact test. Significant *p*-values (<0.01). LARN: Reference Levels of Nutrients and Energy Intake. MNs: macronutrients.

**Table 4:** Odds ratios (ORs) and 95% confidence intervals (CIs) for the severity of IBS-symptoms with dietary habits.

	Adjusted OR	95% CI	*p*-Value
Mediterranean Diet (MD)	1.75	0.73–41.86	0.73
Carbohydrate intake	0.73	0.06–8.43	0.80
Lipid intake	0.67	0.07–6.77	0.73
Protein intake	0.81	0.004–1.81	0.11

Data were adjusted for age, gender, body mass index and energy.

## Data Availability

All sequencing data associated with this study were uploaded to the NCBI bioproject database: PRJNA391149 (https://www.ncbi.nlm.nih.gov/bioproject/?term=PRJNA391149).

## Supplementary Materials

The following are available online at https://www.mdpi.com/2072-6643/13/5/1479/s1, Supplementary Figure S1: Alpha and beta diversity analyses of IBS patients. Supplementary Figure S2: Alpha and beta diversity analyses of CTRL subjects. Supplementary Figure S3: Alpha and beta diversity analyses of CTRLs vs. IBS (LARN groups). Supplementary Figure S4: Alpha and beta diversity analyses of CTRLs vs. IBS (MNs groups and non-MNs groups). Table S1: Mediterranean diet–food frequency intake.
