# Supplementary material for: Association between Dietary Habits and Fecal Microbiota Composition in Irritable Bowel Syndrome Patients: A Pilot Study
Source: Nutrients. 2021 Apr 27;13(5):1479. doi: 10.3390/nu13051479 (PMC8170891; doi:10.3390/nu13051479)
Supplement: Supplementary file 1 [file nutrients-13-01479-s001.zip › nutrients-1144269-supplementary.pdf]

Supplementary Material

A: IBS CARBOHYDRATE INTAKE

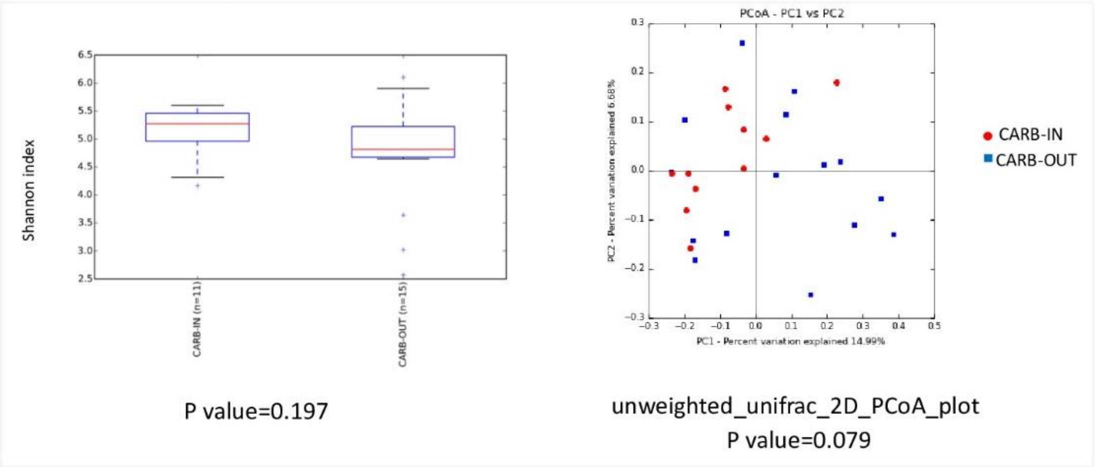

B: IBS LIPID INTAKE

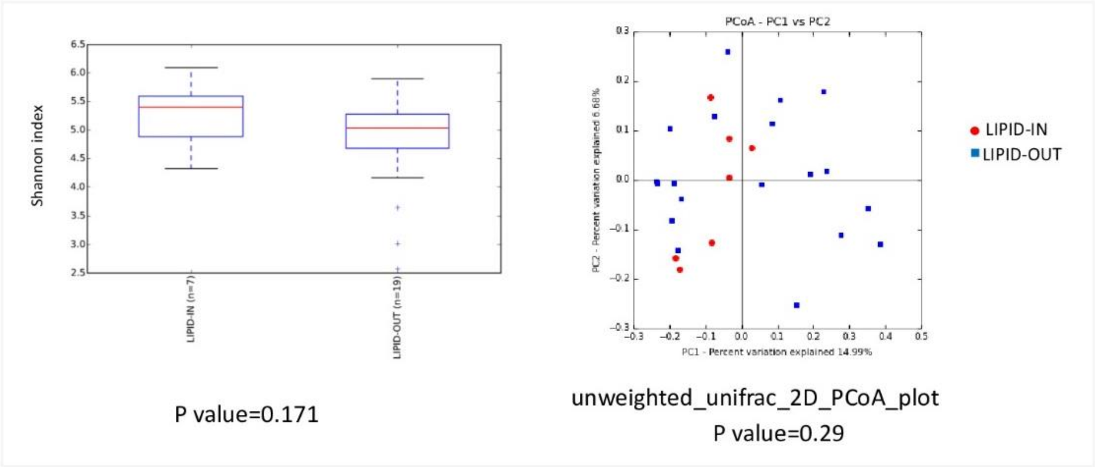

C: IBS PROTEIN INTAKE

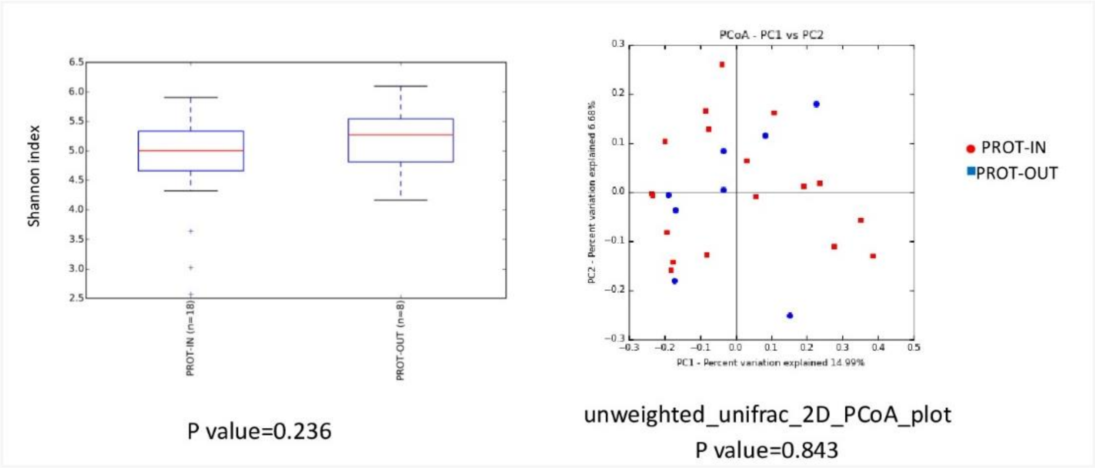

Supplementary Figure S1. Alpha and beta diversity analyses of IBS patients. Boxplots representing alpha diversity (Shannon index). The plots represent the median, 25th, and 75th percentiles calculated for each group. The *p*-values reported in the figure were calculated by nonparametric statistical test. Principal coordinates analyses plots show the first two principal coordinates (axes) for principal coordinates analysis (PCoA) using unweighted UniFrac. The *p*-values reported in the figure were calculated by PERMANOVA analyses. **(A)** IBS Carbohydrate intake. (A1) Shannon index of CARB-in and CARB-out groups; (A2) Principal coordinates analysis plot of CARB-in and CARB-out groups. **(B)** IBS fat intake. (B1) Shannon index of LIPID-in and LIPID-out groups; (B2) Principal coordinates analysis plot of LIPID-in and LIPID-out groups. **(C)** IBS protein intake. (C1) Shannon index of PROT-in and PROT-out groups. (C2) Principal coordinates analysis plot of PROT-in and PROT-out groups.

A: CTRL CARBOHYDRATE INTAKE

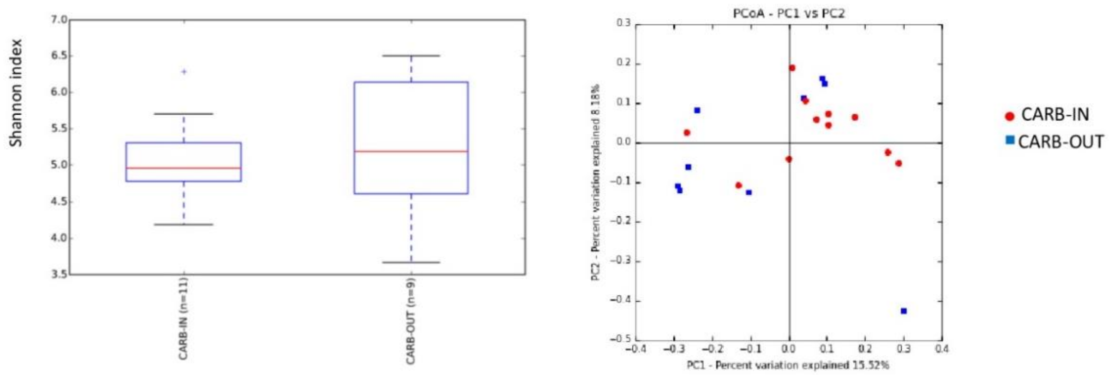

P value=0.537

unweighted_unifrac_2D_PCoA_plot

P value=0.92

B: CTRL LIPID INTAKE

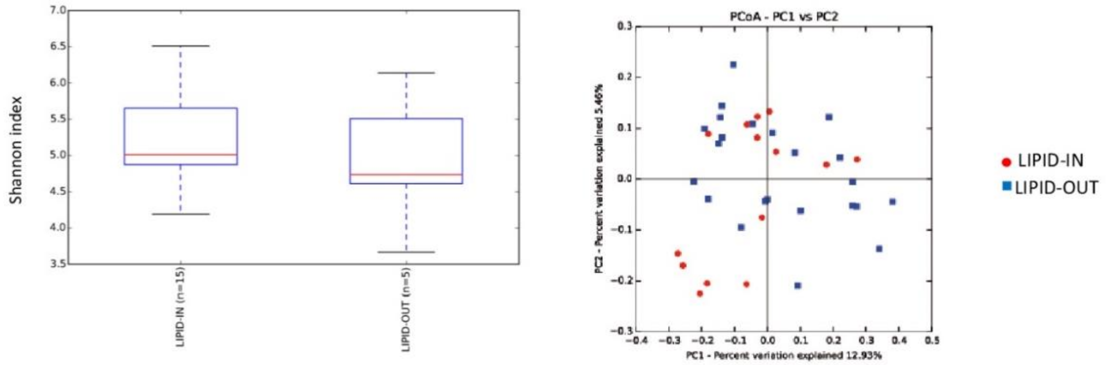

P value from T TEST: 0.485

unweighted_unifrac_2D_PCoA_plot

P value from PERMANOVA TEST: 0.842

C: CTRL PROTEIN INTAKE

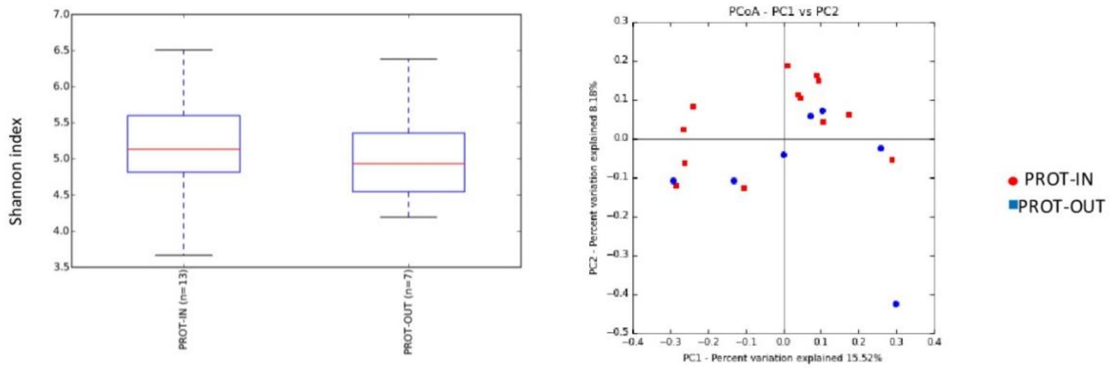

P value from T TEST: 0.66

unweighted_unifrac_2D_PCoA_plot

P value from PERMANOVA TEST: 0.23

Supplementary Figure S2. Alpha and beta diversity analyses of CTRL subjects. Boxplots representing alpha diversity Shannon index. The plots represent the median, 25th, and 75th percentiles calculated for each group. The *p*-values reported in the figure were calculated by nonparametric statistical test. Principal coordinates analysis plots show the first two principal coordinates (axes) for principal coordinates analysis (PCoA) using unweighted UniFrac. The *p*-values reported in the figure were calculated by PERMANOVA analyses. **(A)** CTRL carbohydrate intake. (A1) Shannon index of CARB-in and CARB-out groups; (A2) Principal coordinates analysis plot of CARB-in and CARB-out groups. **(B)** CTRL fat intake. (B1) Shannon index of LIPID-in and LIPID-out groups; (B2) Principal coordinates analysis plot of LIPID-in and LIPID-out groups. **(C)** CTRL protein intake. (C1) Shannon index of PROT-in and PROT-out groups; (C2) Principal coordinates analysis plot of PROT-in and PROT-out groups.

A. CARBOHYDRATE INTAKE

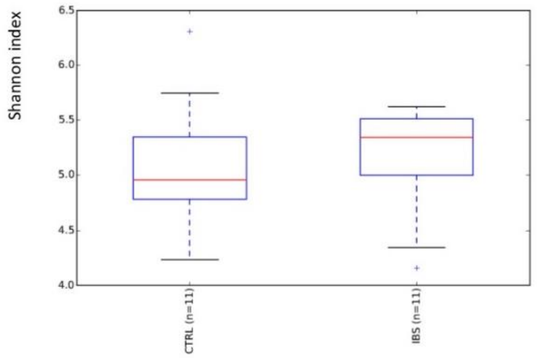

P value=0.662

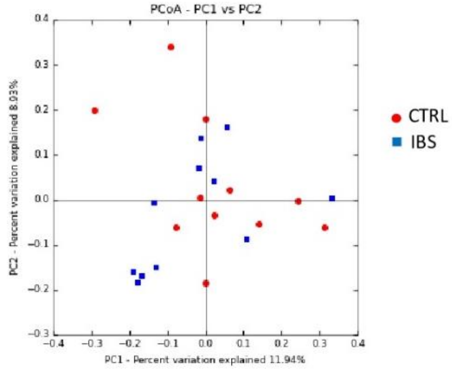

unweighted_unifrac_2D_PCoA_plot

P value=0.47

B: LIPID INTAKE

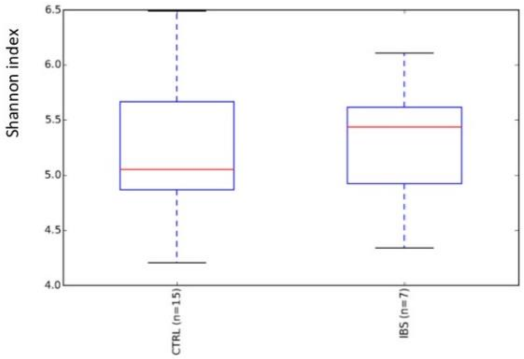

P value=0.831

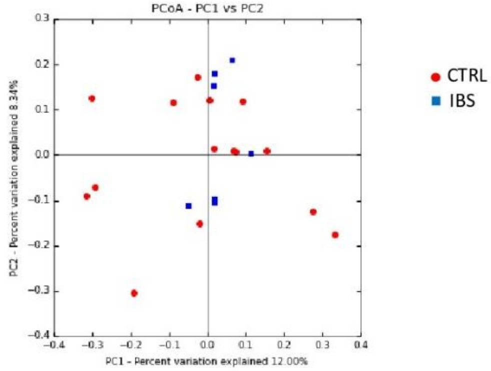

unweighted_unifrac_2D_PCoA_plot

P value=0.73

C: PROTEIN INTAKE

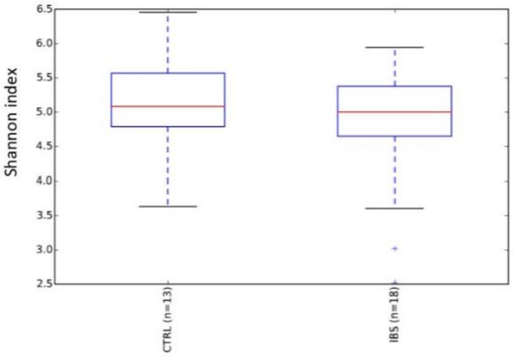

P value=0.192

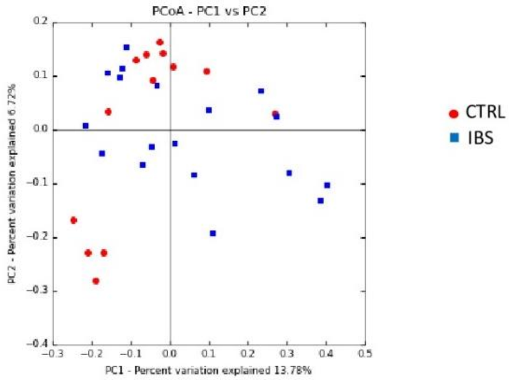

unweighted_unifrac_2D_PCoA_plot

P value=0.232

Supplementary Figure S3. Alpha and beta diversity analyses of CTRLs vs. IBS (LARN groups). Boxplots representing alpha diversity Shannon index. The plots represent the median, 25th, and 75th percentiles calculated for each group. The *p*-values reported in the figure were calculated by nonparametric statistical test. Principal coordinates analysis plots show the first two principal coordinates (axes) for principal coordinates analysis (PCoA) using unweighted UniFrac. The *p*-values reported in the figure were calculated by PERMANOVA analyses. **(A)** CTRL vs. IBS carbohydrate intake. (A1) Shannon index of IBS and CTRL groups; (A2) Principal coordinates analysis plot of IBS and CTRL groups. **(B)** CTRL vs IBS fat intake. (B1) Shannon index of IBS and CTRL groups; (B2) Principal coordinates analysis plot of IBS and CTRL groups. **(C)** CTRL vs. IBS Protein intake. (C1) Shannon index of IBS and CTRL groups; (C2) Principal coordinates analysis plot of IBS and CTRL groups.

A: MNs GROUPs

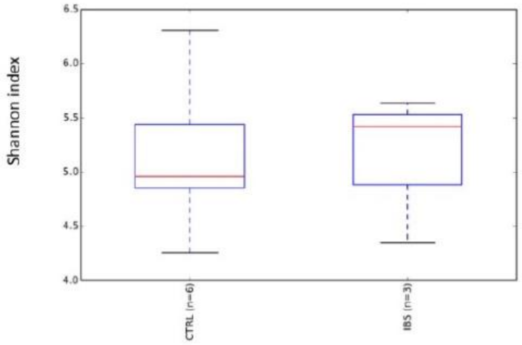

P value=0.972

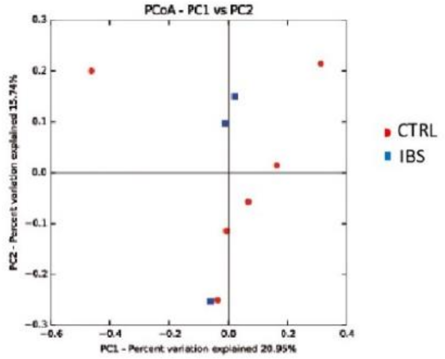

unweighted_unifrac_2D_PCoA_plot

P value=0.682

B: non-MNs GROUPs

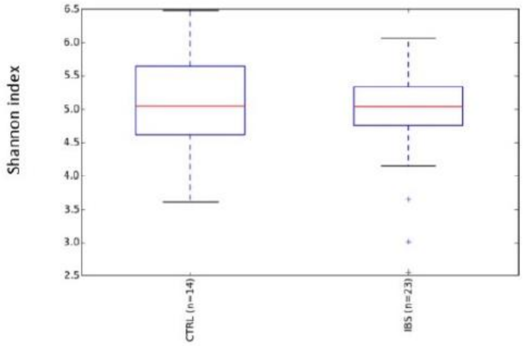

P value=0.414

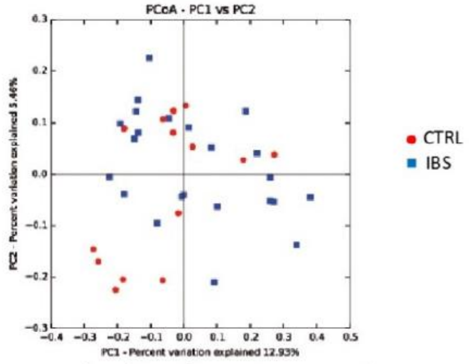

unweighted_unifrac_2D_PCoA_plot

P value=0.651

C: CTRL “MNs GROUP” vs. IBS “non-MNs GROUP”

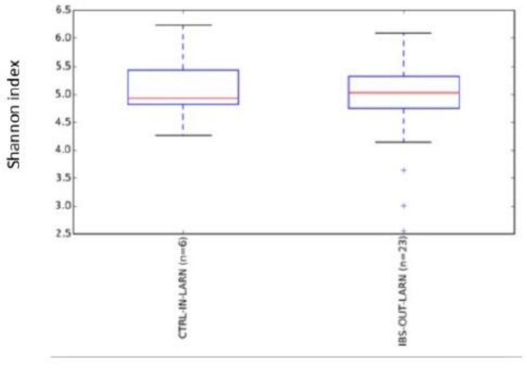

P value=0.523

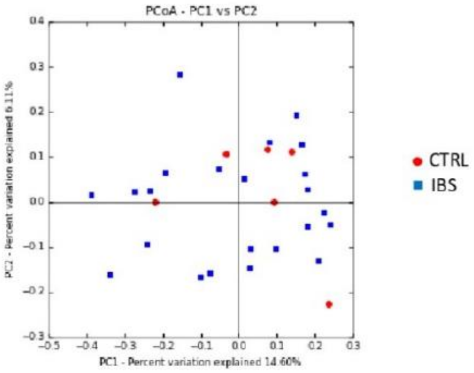

unweighted_unifrac_2D_PCoA_plot

P value=0.543

Supplementary Figure S4. Alpha and beta diversity analyses of CTRLs vs. IBS (MNs groups & non-MNs groups). Boxplots representing alpha diversity Shannon index. The plots represent the median, 25th, and 75th percentiles calculated for each group. The *p*-values reported in the figure were calculated by nonparametric statistical test. Principal coordinates analysis plots show the first two principal coordinates (axes) for principal coordinates analysis (PCoA) using unweighted UniFrac. The *p*-values reported in the figure were calculated by PERMANOVA analyses. **(A)** in-LARN intake. (A1) Shannon index of IBS and CTRL groups; (A2) Principal coordinates analysis plot of IBS and CTRL groups. **(B)** out-LARN intake. (B1) Shannon index of IBS and CTRL groups; (B2) Principal coordinates analysis plot of IBS and CTRL groups. **(C)** CTRL in-LARN vs IBS out-LARN intake. (C1) Shannon index of IBS and CTRL groups; (C2) Principal coordinates analysis plot of IBS and CTRL groups.

Supplementary Table S1: Mediterranean diet - Food frequency intake.

Intake	IBS (N=28)	CTRL (N=21)	*p*-value
Fruit, *1-2 portions/day*	7 (25%)	10 (48%)	0.13
Vegetables, *≥2 portions/meal*	11 (39%)	15 (71%)	**0.04**
Cereals, *1-2 portions/meal*	25 (89%)	19 (90%)	>0.9
Potatoes, *≤3 portions/week*	24 (86%)	20 (95%)	0.38
Olive oil, *1 portion/meal*	21 (75%)	19 (90%)	0.27
Walnuts, *1-2 portions/day*	0 (0%)	6 (29%)	**0.004**
Milk – yogurt – cheeses, *2 portions/day*	15 (54%)	18 (86%)	**0.03**
Legumes, *≥2 portions/week*	6 (21%)	5 (24%)	>0.9
Eggs, *2-4 portions/week*	15 (54%)	11 (52%)	>0.9
Fish and seafood, *≥2 portions/week*	12 (43%)	17 (81%)	**0.009**
White meat (poultry), *2 portions/week*	17 (61%)	15 (71%)	0.55
Red meat and sausages, *<2 portions/week*	12 (43%)	13 (62%)	0.25
Desserts, *≤2 portions/week*	8 (26%)	5 (24%)	0.75
Fermented drinks, *1-2 glasses/day*	2 (7%)	6 (29%)	0.06

The food frequency intake according to the MD adherence-degree from Monteagudo *et al*. (39). Data were calculated by the Fisher's exact test. Significant *p*-values (<0.05).
